# MCL1 inhibition to enhance the efficacy of MYB targeting in pediatric acute myeloid leukemia

**DOI:** 10.1038/s41419-026-08847-2

**Published:** 2026-05-13

**Authors:** Alexia Tsakaneli, Noelia Che, Clemence Virely, Bryony McCord, Luca Gasparoli, Aerin Loe, Kent Fung, Olivia Ciampa, Nuriye Meryem Alver, Qingyin Li, Jack Bartram, David O’Connor, Marc Mansour, Owen Williams

**Affiliations:** 1 Cancer Section, Developmental Biology and Cancer Department, UCL Great Ormond Street Institute of Child Health, London, UK; 2 Department of Haematology, Cancer Institute, University College London, London, UK; 3 Department of Paediatric Haematology, Great Ormond Street Hospital for Children, London, UK

**Keywords:** Targeted therapies, Target validation, Acute myeloid leukaemia

## Abstract

Outcomes for pediatric acute myeloid leukemia (AML) have improved significantly in recent years. However, relapsed and refractory disease remains a significant problem. The chemotherapy burden experienced by these patients makes the translational development of non-genotoxic experimental therapies attractive. We previously reported that the anti-helminth drug mebendazole induces degradation of the transcription factor MYB and has potent anti-AML activity. In the present study, we use CRISPR drop-out screening to identify genes encoding the proapoptotic regulators BAK and NOXA as hits conferring resistance to mebendazole activity in AML cells. Conversely, targeting MCL1 with a BH3-mimetic significantly enhanced the anti-AML activity of mebendazole in both AML cell lines in vitro and pediatric patient-derived xenograft (PDX) AML cells ex vivo. Treatment of mice transplanted with THP-1 AML cells or aggressive infant PDX AML cells with this drug combination significantly impaired disease progression in vivo. Our data indicate that mebendazole-induced MYB degradation in combination with MCL1 targeting is a novel non-genotoxic therapeutic strategy for pediatric AML.

## Introduction

Outcomes in pediatric leukemia overall have improved dramatically in recent times by a combination of intensive chemotherapy, risk stratification and bone marrow transplantation. However, pediatric Acute Myeloid Leukaemia (AML) remains a challenging disease with post-chemotherapy disease relapse being a significant problem and overall survival plateauing at 70–75% of diagnosed cases [1]. Further intensification of chemotherapy is unlikely to provide benefit due to treatment-related toxicities [2]. In this context, advances in our understanding of disease mechanisms and associated transcriptional networks have uncovered leukemia susceptibilities with promising potential for translation into novel therapies.

The transcription factor MYB is essential for normal hematopoiesis [3]. However, leukemia cells exhibit an exaggerated dependence on transcriptional programs orchestrated by MYB, suggesting the existence of a therapeutic window for its targeting, particularly in AML [3, 4]. In common with other transcription factors, the protein structure of MYB offers few possibilities for direct pharmacological inhibition. In contrast, several studies have demonstrated the possibility of targeting MYB indirectly, with particularly promising approaches based on inhibition of binding to transcriptional co-factors essential for MYB function in leukemia [5–8].

In a previous study, we reported that MYB could be targeted through induced proteasomal degradation by exposure of AML cells to the anti-helminth drug mebendazole [9]. Mebendazole was rationally identified by connectivity map analysis using an oncogenic MYB gene expression signature derived from a conditional mouse model of AML [10]. This drug was shown to induce rapid degradation of MYB in AML cells representing a range of different molecular subtypes, resulting in potent anti-AML activity. More recently, the anti-AML activity of mebendazole has been confirmed independently [11–14], albeit via distinct molecular mechanisms in some reports [13]. In this regard, it is important to note that overexpression of a stabilised MYB mutant protein that retains transcriptional activity was able to partially rescue inhibition of leukemia self-renewal by mebendazole in our study [9]. This demonstrates that the short half-life of MYB protein, coupled with its essential function in AML, makes these cells particularly vulnerable to disruption of its chaperone-mediated turnover [9]. Despite its undoubted pleiotropic effects, mebendazole can therefore be used as a MYB-directed therapeutic in AML.

Mebendazole has been shown to synergise with conventional anti-AML chemotherapeutics [13], suggesting that it could be incorporated into current treatment. However, its combination with non-genotoxic therapies has not been addressed. In the present study, we demonstrate that inhibition of the anti-apoptotic BCL-2 family member MCL1 enhances the anti-AML activity of mebendazole. Given the clinical impact of apoptosis-based therapies [15], this novel targeting strategy offers a translational opportunity in pediatric AML.

## Results

**Fig. 1:**
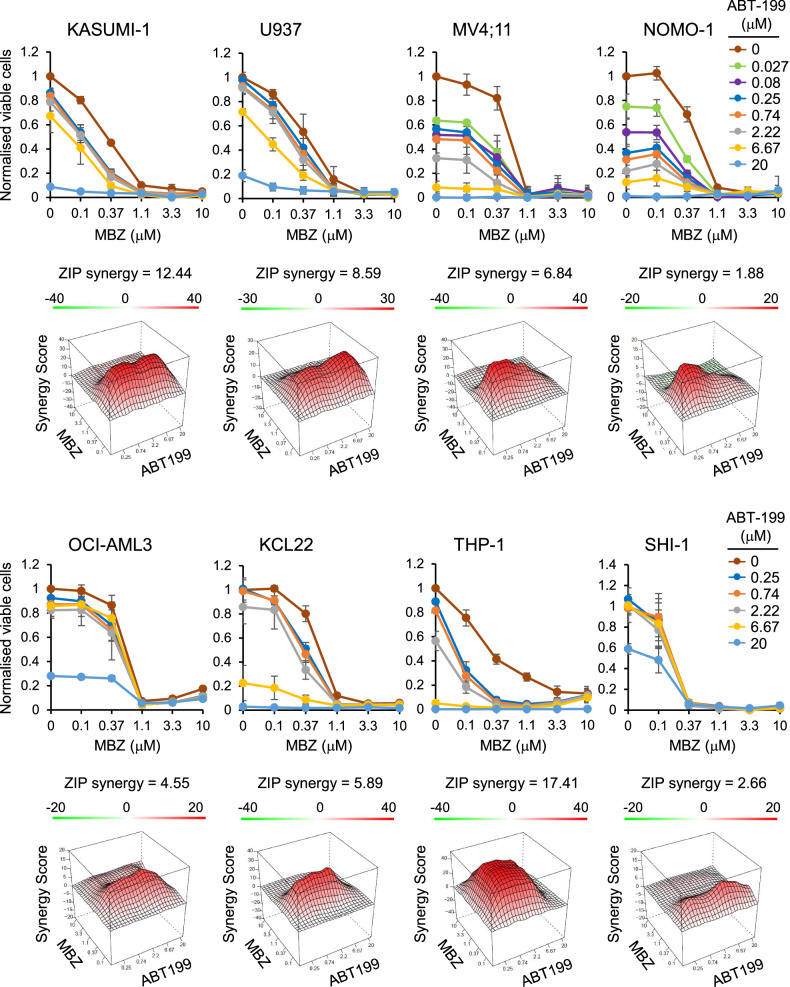
Anti-AML activity of BCL-2 inhibition combined with mebendazole. Plots show viability of AML cell lines following 72 h exposure to indicated concentrations of ABT-199 and mebendazole (MBZ) (upper panels), and 3D synergy maps and ZIP synergy scores (lower panels) of data calculated with SynergyFinder (version 2.0). Graph points and error bars represent means and SD of *n* = 3 independent experiments.

AML has been shown to be sensitive to modulation of anti-apoptotic BCL-2 family members in both the pre-clinical and clinical settings [16]. In particular, the BH3-mimetic venetoclax (ABT-199), which targets BCL-2, has been approved in combination with hypomethylating agents for the treatment of newly diagnosed adult AML patients not eligible for conventional intensive chemotherapy [16, 17]. ABT-199 has also shown efficacy in pediatric AML and is currently in a phase 3 clinical trial for relapsed and refractory disease (NCT05183035). Importantly, MYB has been shown to bind the *BCL2* locus and positively regulate *BCL2* transcription in AML cells [7, 18]. This implied that mebendazole may also regulate BCL-2 expression through its effect on MYB degradation. Exposure of THP-1 AML cells to mebendazole for 24 h resulted in significant inhibition of BCL2 gene and protein expression (Supplementary Fig. S1A, B). In contrast, mebendazole increased the expression of the anti-apoptotic BCL-2 family member MCL1 (Supplementary Fig. S1, C). This suggested that AML cells may exhibit enhanced susceptibility to the combination of mebendazole with ABT-199. Indeed, exposure of an AML cell line panel to mebendazole and ABT-199 increased anti-AML activity in all cell lines tested in comparison to either drug alone, demonstrating additive to synergistic combination effects (Fig. 1). However, it should be noted that some cell lines were relatively resistant to ABT-199 treatment on its own, requiring high concentrations for any detectable anti-AML activity.

**Fig. 2:**
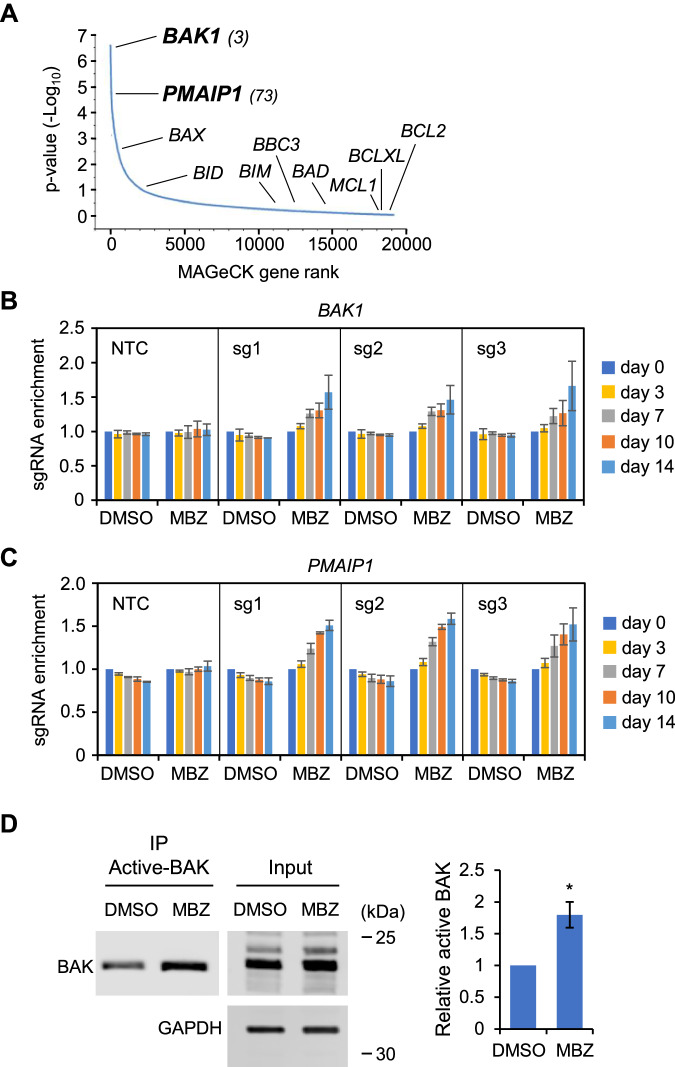
The BAK/NOXA/MCL1 apoptotic pathway regulates mebendazole-induced cell death in AML cells. **A** Graph depicting the significance of positively selected sgRNA, plotted against the MAGeCK gene rank, in transduced THP-1 cells exposed to IC90 mebendazole compared to DMSO-treated cells. Genes targeted by positively selected sgRNA are indicated: *BAK1* (rank 3) and *PMAIP1* (rank 73). Graph of enrichment of lentiviral non-targeting control (NTC) sgRNA and three sgRNA (sg1-3) from the Brunello CRISPR-knockout library targeting **B** *BAK1* or **C** *PMAIP1* in transduced THP-1 cells cultured in DMSO control or 1 μM mebendazole (MBZ) for 14 days. Enrichment of each sgRNA was calculated as the ratio of % transduced (GFP^+^) cells at the indicated day over that at day 0 of the culture. **D** Example of Western blot (left panel) and quantification (right panel) of active BAK immunoprecipitates from THP-1 cells exposed to 1 μM MBZ for 24 h, normalized to DMSO-treated controls. Bars and error bars represent means and SD of *n* = 3 independent experiments. *P < 0.05, two-tailed one-sample *t* test.

To determine whether any additional pathways regulating cell death could sensitise AML cells to mebendazole, we then performed a genome-wide CRISPR-Cas9 dropout screen. THP-1 AML cells were exposed to 1 μM mebendazole or DMSO for 10 days (Fig. 2A). This concentration of mebendazole resulted in approximately 90% cell death over this time course (data not shown). MAGeCK [19] analysis of the screen identified 324 positively and 17 negatively (FDR < 0.05) enriched genes (Supplementary Table S1). Pathway enrichment analysis suggested that ribosomal RNA processing may play a role in mebendazole-induced death (supplementary Fig. S2). In addition, MAGeCK analysis ranked sgRNA targeting *BAK1* and *PMAIP1* genes as the third and seventy-third most positively enriched sgRNAs in THP-1 cells surviving mebendazole treatment, respectively (Fig. 2A). *BAK1* encodes BAK, an essential proapoptotic mediator of the intrinsic cell death pathway, usually bound and sequestered by the antiapoptotic MCL1 and Bcl-xL proteins. *PMAIP1* encodes the BH3-only proapoptotic NOXA protein, which specifically binds and inhibits MCL1 upon induction of apoptosis [20]. We next validated the screen by examining enrichment of 3 sgRNA, obtained from the Brunello library, targeting either *BAK1* or *PMAIP1* upon mebendazole exposure over 14 days (Fig. 2B, C). Mebendazole was found to positively enrich all the sgRNA targeting the proapoptotic genes, but not the non-targeting control (NTC) sgRNA. Furthermore, exposure of THP-1 cells to 1 μM mebendazole for 24 h resulted in BAK activation (Fig. 2D) and apoptosis in response to 48 h mebendazole treatment was dramatically reduced in THP-1 cells transduced with *BAK1* or *PMAIP1* targeting sgRNA (Supplementary Fig. S3). Taken together, these data suggest that in the context of mebendazole-induced BCL-2 suppression, the MCL1/BAK/NOXA apoptosis-regulating pathway may play an important role in AML cell survival.

**Fig. 3:**
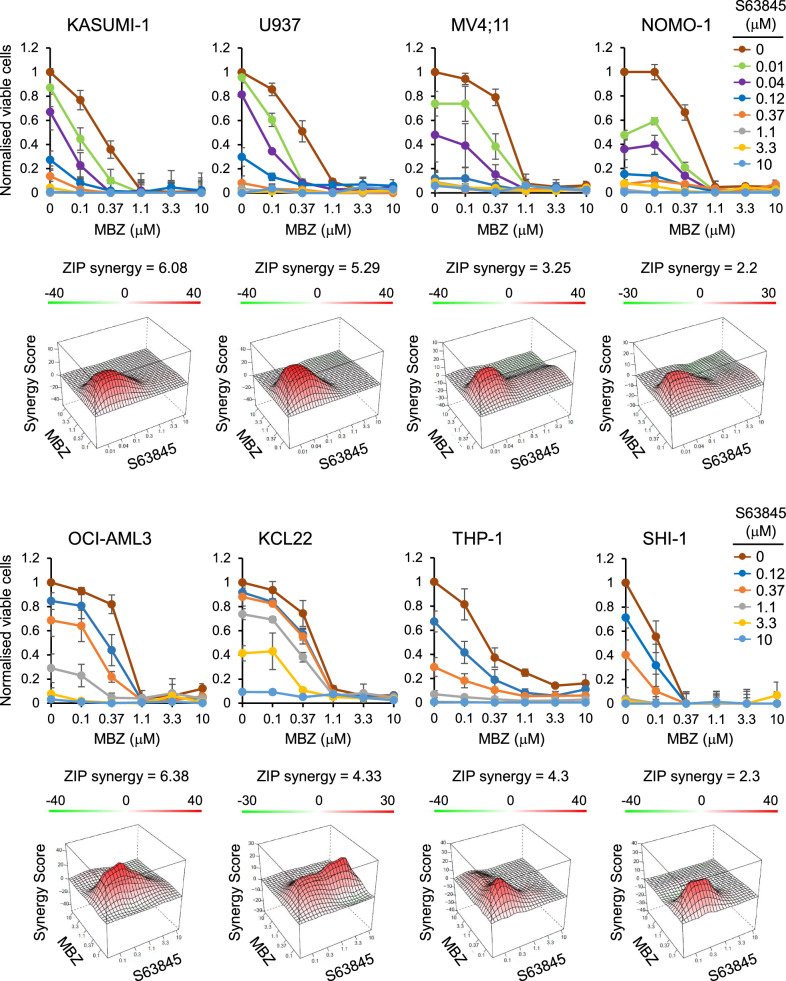
Anti-AML activity of MCL1 inhibition combined with mebendazole. Plots show viability of AML cell lines following 72 h exposure to indicated concentrations of S63845 and mebendazole (MBZ) (upper panels), and 3D synergy maps and ZIP synergy scores (lower panels) of data calculated with SynergyFinder (version 2.0). Graph points and error bars represent means and SD of *n* = 3 independent experiments.

To determine whether inhibition of MCL1 could improve the efficacy of mebendazole activity in AML cells, we then exposed the AML cell line panel to the MCL1-targeting BH3-mimetic S63845 [21], alone and in combination with mebendazole (Fig. 3). Indeed, MCL1 inhibition enhanced the anti-AML activity of mebendazole in all cell lines tested, with ZIP synergy scores in the additive range. It is important to note that whereas some AML cell lines were relatively resistant to ABT-199 treatment alone (Fig. 1), all of them were significantly more sensitive to MCL1 inhibition with S63845 (Fig. 3).

**Fig. 4:**
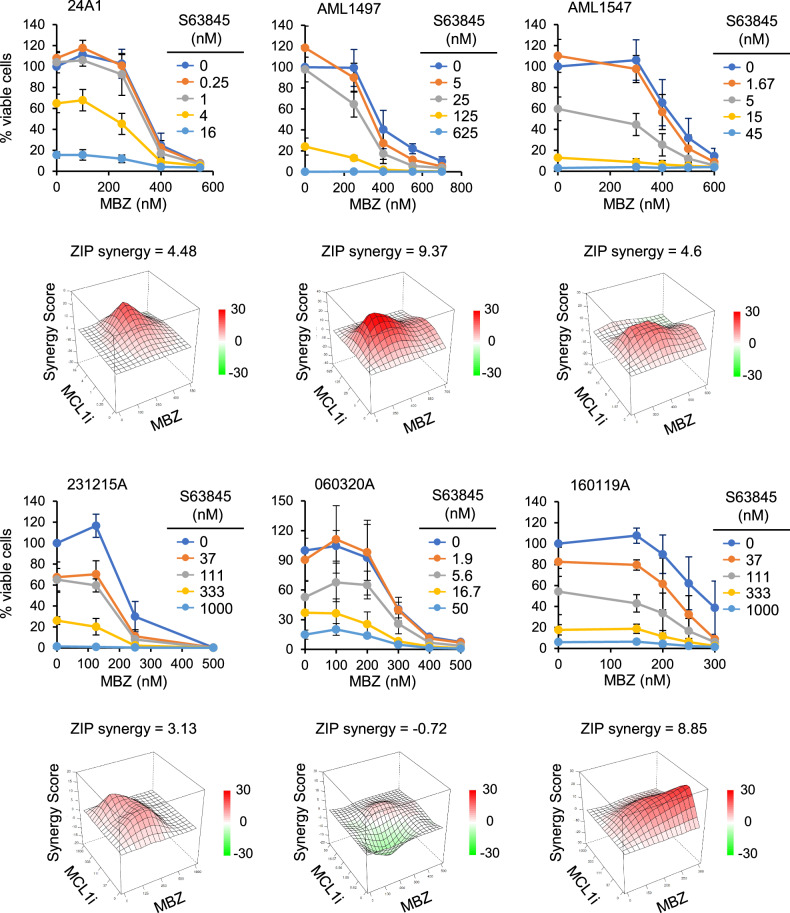
MCL1 inhibition enhances the killing of pediatric PDX AML cells. Plots show viability of PDX AML cells, grown on OP9 monolayers, following 5 days exposure to indicated concentrations of S63845 and mebendazole (MBZ) (upper panels), and 3D synergy maps and ZIP synergy scores (lower panels) of data calculated with SynergyFinder + . Graph points and error bars represent means and SD of *n* = 3 independent experiments.

To further explore the clinical potential of combining mebendazole with BH3-mimetics, we first examined sensitivity to BCL-2 and MCL1 inhibition ex vivo in 6 independent patient-derived xenograft (PDX) leukemia models that we generated from aggressive pediatric AML samples (Supplementary Table S2), consisting of three de novo childhood *KMT2A*r AML (AML1497, AML1547, 231215 A), one relapsed childhood *KMT2A*r AML (24A1), one infant *KMT2A*r AML (060320 A) and one infant monosomy 7 (160119 A). For these experiments, we used a recently described co-culture of AML cells grown on an irradiated OP9 monolayer [22], ensuring that all PDX AML samples proliferated over the course of the experiment. In all samples tested, AML cells exhibited greater sensitivity to S63845 than to ABT-199 (Supplementary Fig. S4). This is consistent with a recent study demonstrating more potent activity of S63845 in comparison to ABT-199 in a range of AML cell lines and primary cells [23]. We then used the same co-culture to examine the sensitivities of the PDX AML panel to combinations of mebendazole with S63845. In all samples, mebendazole enhanced the anti-AML activity of S63845, ZIP synergy scores being in the additive range (Fig. 4), as for the AML cell line panel. In contrast, although both drugs had significant impacts on colony formation by normal human CD34^+^CD133^+^ cord blood cells, significant colony-forming activity remained at drug concentrations that effectively eliminated all PDX AML cells (Supplementary Fig. S5).

**Fig. 5:**
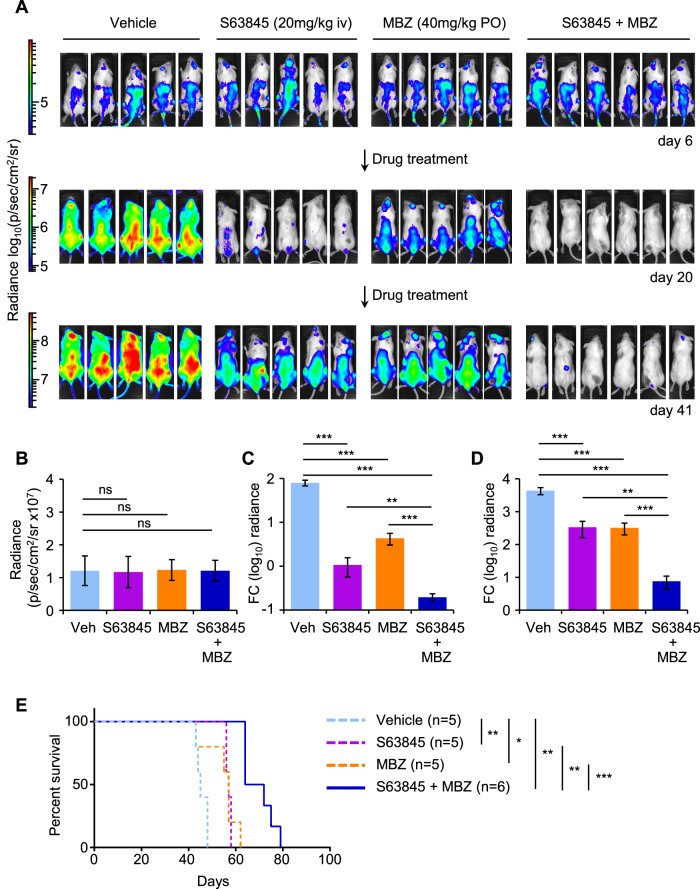
MCL1 inhibition enhances anti-AML activity of mebendazole in vivo. **A** Bioluminescence imaging of NSG recipient mice 6 days after injection with THP-1-LUC2 cells, and before drug treatment, (top), and 20 days (middle) and 41 days (bottom) after treatment with S63845 (MCL1i) and mebendazole (MBZ). Mice were treated twice with S63845 (20 mg/kg, iv) on days 7 and 13 and with MBZ (40 mg/kg, gavage) on days 2, 3, 4, 8, 9, 11, 28, 30 and 32. Bars for luminescence signal represent photons/s/cm^2^/steradian. Luminescence signal in treatment groups, 6 days after THP1-LUC2 cell injection and before drug treatment (**B**), and fold change (FC) in luminescence signal in the groups on days 20 (**C**) and 41 (**D**). Bars and error bars represent means and SD of values from vehicle control (*n* = 5), S63845 (*n* = 5), MBZ (*n* = 5) and drug combination (*n* = 6) treated groups. **P < 0.01; ***P < 0.001; n.s. not significant, unpaired two-tailed Student’s *t* test. **E** Survival curve for the different groups of drug-treated mice. *P < 0.05; **P < 0.01; ***P < 0.001, log-rank test.

**Fig. 6:**
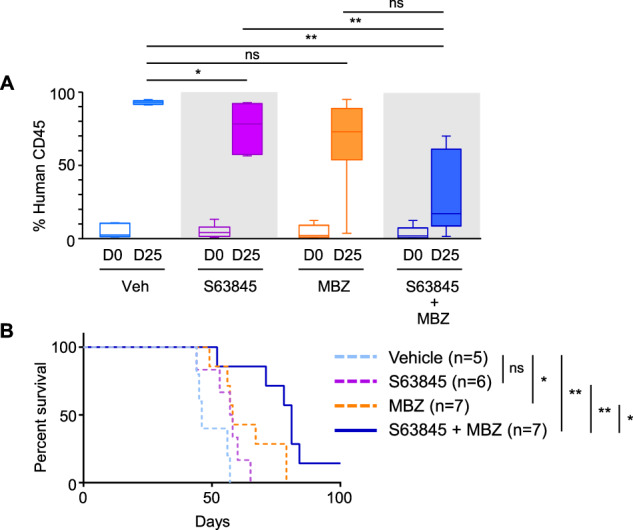
A combination of MCL1 inhibition with mebendazole impairs disease progression in vivo induced by pediatric PDX AML cells. **A** Plot shows the percentage of human CD45^+^ peripheral blood cells (calculated as a percentage of total human + mouse CD45^+^ cells) in NSG mice 76 days after transplantation with an infant *KMT2A*-*MLLT10* PDX AML sample (060320 A) and before drug treatment (day 0) and 25 days after treatment with S63845 (MCL1i) and mebendazole (MBZ). Mice were treated twice with S63845 (20 mg/kg, iv) on days 1 and 8, and with MBZ (40 mg/kg, gavage) on days 2-4, 9-11, 29, 30 and 32. Bars and error bars represent means and SD of values from vehicle control (*n* = 5), S63845 (*n* = 6), MBZ (*n* = 7) and drug combination (*n* = 7) treated groups. *P < 0.05; **P < 0.01; n.s. not significant, unpaired two-tailed Student’s *t* test. **B** Survival curve for the different groups of drug-treated mice. *P < 0.05; **P < 0.01; n.s. not significant, log-rank test.

We previously reported that mebendazole treatment significantly impaired disease progression in NSG mice transplanted with pediatric THP-1 AML cells [9]. To determine whether the effect of mebendazole could be enhanced by combining it with MCL1 targeting, we transplanted NSG mice with luciferase-expressing THP-1 cells, confirmed leukemia engraftment by bioluminescence imaging (Fig. 5A, B) and subjected engrafted mice to treatment with mebendazole and S63845 alone or in combination, or with vehicle control. For this experiment, mebendazole was administered via oral gavage, as recently published [12], rather than mixed into the diet, as in our original study. Both mebendazole and S63845 treatment impaired disease progression when administered alone and were significantly more effective when the drugs were combined (Fig. 5A–D). Disease latency reflected bioluminescence monitoring of disease progression, with each drug on its own able to prolong the survival of transplanted mice and combination treatment significantly more effective (Fig. 5E). Next, we examined whether the in vivo efficacy of this drug combination would be replicated using PDX AML. For this experiment, we used the infant *KMT2A-MLLT1*0 PDX AML sample (060320A) in which we could detect disease progression by the emergence of AML cells into the peripheral blood. Engraftment of NSG mice was confirmed by peripheral blood sampling 75 days after transplantation, following which mice were treated with mebendazole and S63845 alone or in combination (Fig. 6). Although effects on disease progression were more heterogeneous than in the THP-1 AML model, both drugs demonstrated anti-AML activities and were more effective in combination (Fig. 6A). This was confirmed by analysis of disease latency, with combination therapy being more effective at prolonging survival of transplanted mice (Fig. 6B).

## Discussion

Our data show that inhibition of the anti-apoptotic regulator MCL1 enhances the sensitivity of AML cells to the MYB-targeting drug mebendazole. Furthermore, combination therapy with S63845 and mebendazole was superior to either drug alone at impairing AML disease progression in vivo. Enhanced sensitivity was also seen in AML cell lines exposed to the BCL-2 BH3-mimetic ABT-199 in combination with mebendazole. However, both AML cell lines and pediatric PDX AML samples were more sensitive to S63845 than ABT-199 exposure, confirming recently published data [23].

Elucidation of the molecular mechanisms and pathways governing cell survival has revealed the critical role of apoptotic regulatory machinery in normal developmental processes and in cancer [24]. Alteration of the balance between pro- and anti-apoptotic regulators emerged as a promising strategy for non-genotoxic cancer therapy [25]. BCL-2 targeting with venetoclax has made a major impact on adult AML therapy in combination with hypomethylating agents [16, 17]. This is a particularly effective therapeutic approach in patients not eligible for conventional chemotherapy. However, pre-existing and acquired resistance to venetoclax is a significant problem [26]. The basis for such resistance is the subject of intensive research, given the impact of venetoclax on AML therapy. For example, loss or mutation of *TP53* has been shown to confer venetoclax resistance in preclinical and clinical studies on adult AML [27, 28]. Our AML cell line panel contained two cell lines that were wild-type for *TP53* (OCI-AML3 and MV4;11), with the rest being either *TP53* mutant or null. However, p53 status did not correlate with venetoclax sensitivity, likely due to additional mechanisms of resistance. It is important to note that AML cell line sensitivity to MCL1 inhibition, alone or in combination with mebendazole, did not correlate with p53 status either, although we could not evaluate this for our pediatric PDX AML panel since this did not contain any samples with *TP53* alterations. AML differentiation status was reported to determine sensitivity to therapies incorporating venetoclax, with myelomonocytic and monocytic AML subtypes exhibiting resistance [29, 30]. Such resistance was due to a greater dependence of these AML cells on the anti-apoptotic activity of MCL1 rather than BCL-2. However, a correlation between AML differentiation status and response to venetoclax therapy was not found in subsequent clinical studies [31, 32]. More recently, it was reported that the clinical response to venetoclax therapy is determined by the relative dependence on BCL-2 versus MCL1 and BCL-xL of the leukemic stem cell (LSC) compartment rather than that of the bulk AML blasts [33]. Measurement of the relative expression of these anti-apoptotic proteins in AML LSC offers the possibility of predicting clinical responses to venetoclax-containing therapies [33], as well as using alternative strategies such as MCL1 inhibition. Another approach to such prediction was investigated in a prospective phase 2 clinical trial published this year [34]. This trial demonstrated that analysis of ex vivo AML blast responses to venetoclax was effective at predicting clinical responses to venetoclax in combination with hypomethylating therapy. Interestingly, this study reported that flow cytometric analysis of AML cell viability following exposure to venetoclax was a more accurate predictor of clinical responses than metabolic viability assays [34]. In this context, it is important to note that the greater sensitivity to MCL1 inhibition by S63845 in comparison to BCL-2 inhibition with ABT-199 in our PDX AML samples was determined using ex vivo flow cytometric viability assessment. This suggests that combining mebendazole with MCL1 inhibition rather than with BCL-2 inhibition may be more clinically relevant for pediatric AML therapy.

The global CRISPR drop-out screen established that loss of proapoptotic regulators *BAK1* and *PMAIP1* conferred resistance to mebendazole treatment. The association of these regulators with apoptosis triggered by MCL1 inhibition suggests a role for the MCL1/NOXA/BAK axis in AML cell responses to mebendazole exposure. The potential therapeutic benefit of MCL1 inhibition in AML therapy is widely recognised but has been tempered by unacceptable cardiotoxicity observed in recent clinical trials. The lack of similar toxicities in pre-clinical in vivo models may be explained by the lower affinities of MCL1 inhibitors such as S63845 for mouse versus human MCL1 [35]. Various strategies are being evaluated to overcome this obstacle, including careful dosing of MCL1 inhibitors, to determine whether a therapeutic window exists without triggering toxicities, and indirect MCL1 inhibition through targeting pathways responsible for regulating MCL1 protein levels [20]. An alternative approach was recently reported with the development of novel highly selective MCL1 inhibitors associated with rapid systemic clearance and consequently reduced toxic side-effects [36]. These efforts indicate that despite recent setbacks, MCL1 targeting remains a translationally viable strategy for novel AML therapy. Our data indicate that it can be combined with MYB targeting in a non-genotoxic therapeutic approach with promising pre-clinical activity in pediatric AML.

## Materials and methods

### Mice

NOD-SCID-γ^–/–^ (NSG; The Jackson Laboratory, Bar Harbor, ME, USA) mice were maintained in the UCL GOS ICH animal facilities and experiments were performed according to and approved by the United Kingdom Home Office regulations and followed UCL GOS ICH institutional guidelines.

### AML PDX samples

Ethical approval was given (Research Ethics Committee references 14/EM/0134 and 15225/001) for the use of appropriately consented material from patients with AML at Great Ormond Street Hospital for Children (London, UK) and at other hospitals in the UK. For initial PDX generation, 1–2 × 10^6^ mononuclear cells (Supplementary Table S2) were intravenously injected into 2 G irradiated 5- to 12-week-old female NSG mice. Recipient mice were sacrificed upon developing clinical signs of disease. Human PDX AML cells were harvested and purified from bone marrow using the mouse cell depletion kit (Miltenyi Biotec, Surrey, UK).

### Cell culture and reagents

Human AML cell lines were purchased from the ATCC (THP1) and DSMZ (KASUMI-1, U937, NOMO-1, OCI-AML3, KCL22, SHI-1 and MV4;11), authenticated by short tandem repeat profiling using the PowerPlex 16 system (Promega, Southampton, UK), and mycoplasma-negative status confirmed using the MycoAlert Mycoplasma Detection Kit (Lonza, Verviers, Belgium). The following reagents were used: mebendazole (Merck Life Science UK, Dorset, UK), S63845 (Cayman Chemical Company, MI, USA and Stratech Scientific, Ely, UK) and Venetoclax (ABT-199, Cayman Chemical Company).

### Synergy experiments

Synergy was calculated with the ZIP method using SynergyFinder 2.0 (for AML cell lines) and SynergyFinder+ (for PDX AML cells) (https://synergyfinder.org) [37].

### CRISPR drop-out screen

The mebendazole CRISPR drop-out screen and sequencing is described in Supplementary Methods.

### In vivo transplantation

To examine combination drug treatment in vivo, luciferase-expressing THP-1 cells or PDX AML cells were transplanted into non-irradiated NSG mice. THP-1 transplanted mice were imaged using the IVIS® Lumina Series III (PerkinElmer, Beaconsfield, UK) and PDX AML transplanted mice were monitored by peripheral blood sampling. After engraftment was confirmed, mice were randomly allocated to control or drug-treated groups by flipping a coin. Mebendazole (40 mg/kg, in soybean oil) was administered via gavage and S63845 (20 mg/kg, in 2% w/v TPGS, Merck, in PBS) via intravenous injection. No blinding was used.

### Western blot and immunoprecipitation (IP) analysis

Western blot and IP analyses were performed as detailed in Supplementary Methods. Full-length Western Blots are included in the Supplementary Material.

### Statistics

Statistical significance was determined using Prism 7.0 (GraphPad) software. Statistical analysis of means was performed using the one-sample *t* test or unpaired Student’s *t* test, two-tailed *P* values < 0.05 being considered statistically significant. Variance was similar between groups. Statistical analysis of survival curves was performed using the log-rank test.

## Supplementary information

Supplementary Table S1

CDDIS-25-5224-T aj-checklist

Original Full Western Blots

Revised Clean Tsakaneli et al Supplementary Material

## Data Availability

The data generated in this study are available within the article and its Supplementary data files. The CRISPR drop-out sequencing data generated in this study are publicly available in the Gene Expression Omnibus at GSE293063.
