# Supplementary figures and images for: MCL1 inhibition to enhance the efficacy of MYB targeting in pediatric acute myeloid leukemia
Source: Cell Death Dis. 2026 May 13;17(1):616. doi: 10.1038/s41419-026-08847-2 (PMC13338407; doi:10.1038/s41419-026-08847-2)

Figure 2D

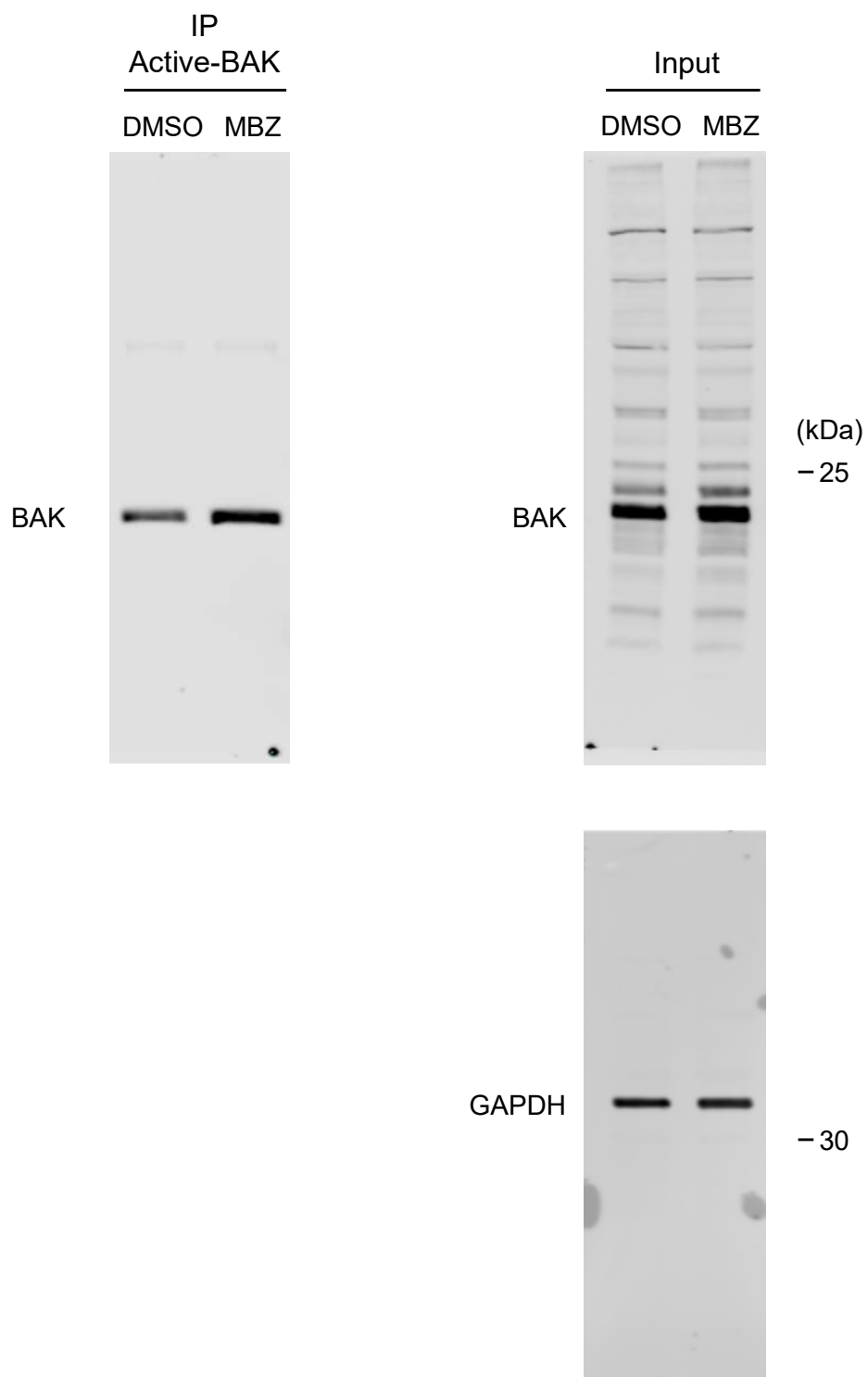

## Supplementary Figure S1

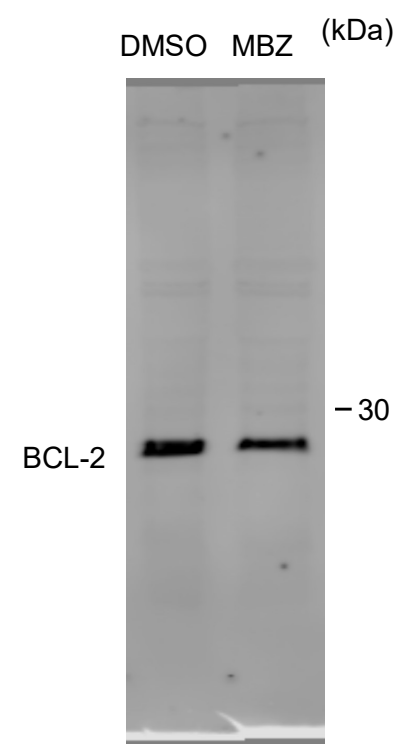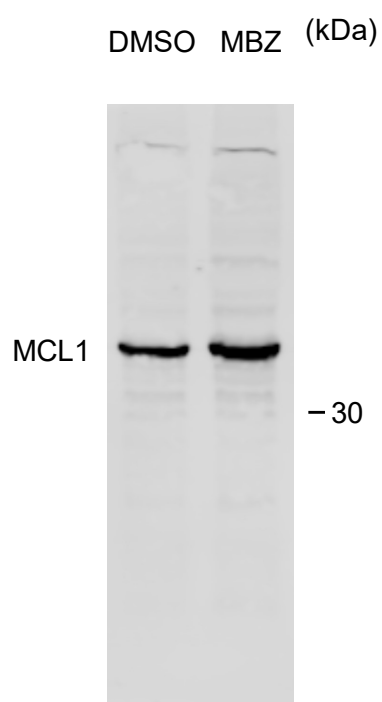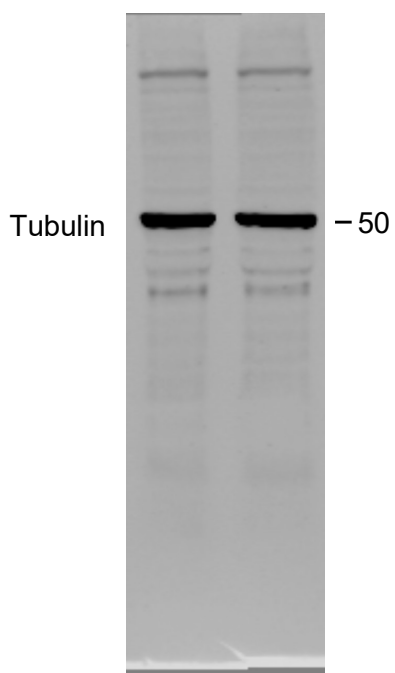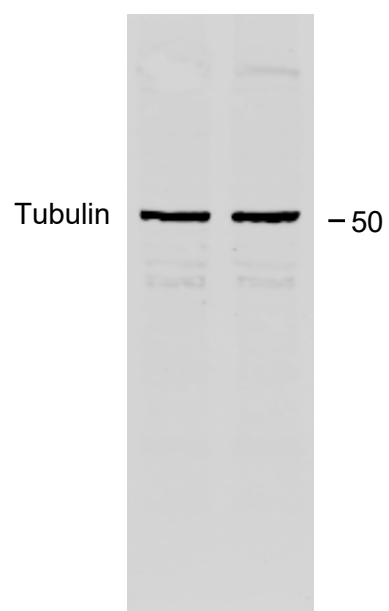

Supplement: Supplementary file 3 — Original Full Western Blots [file 41419_2026_8847_MOESM3_ESM.pdf]
