# Supplementary material for: MCL1 inhibition to enhance the efficacy of MYB targeting in pediatric acute myeloid leukemia
Source: Cell Death Dis. 2026 May 13;17(1):616. doi: 10.1038/s41419-026-08847-2 (PMC13338407; doi:10.1038/s41419-026-08847-2)
Supplement: Supplementary file 4 — Revised Clean Tsakaneli et al Supplementary Material [file 41419_2026_8847_MOESM4_ESM.pdf]

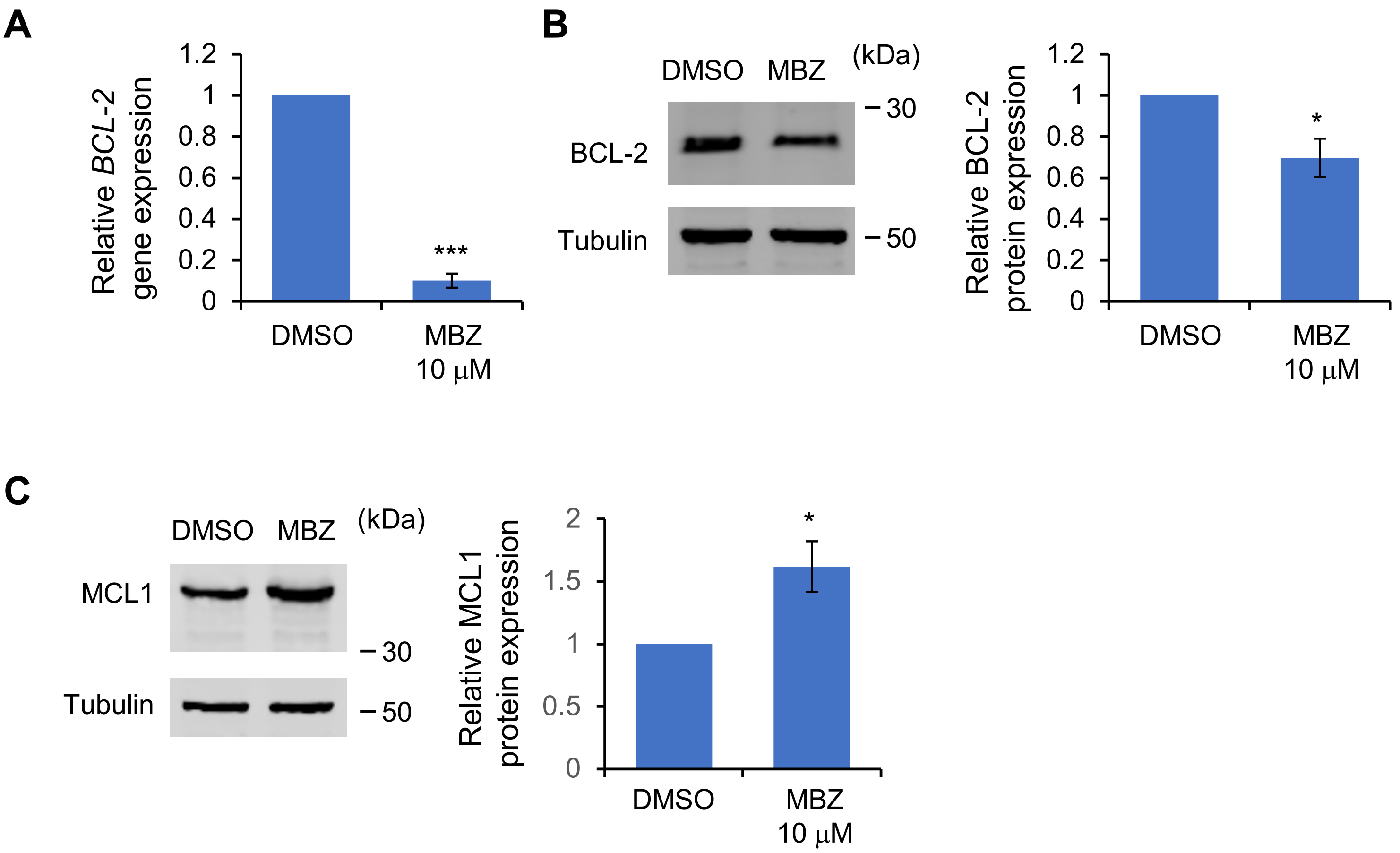

Supplementary Figure S1

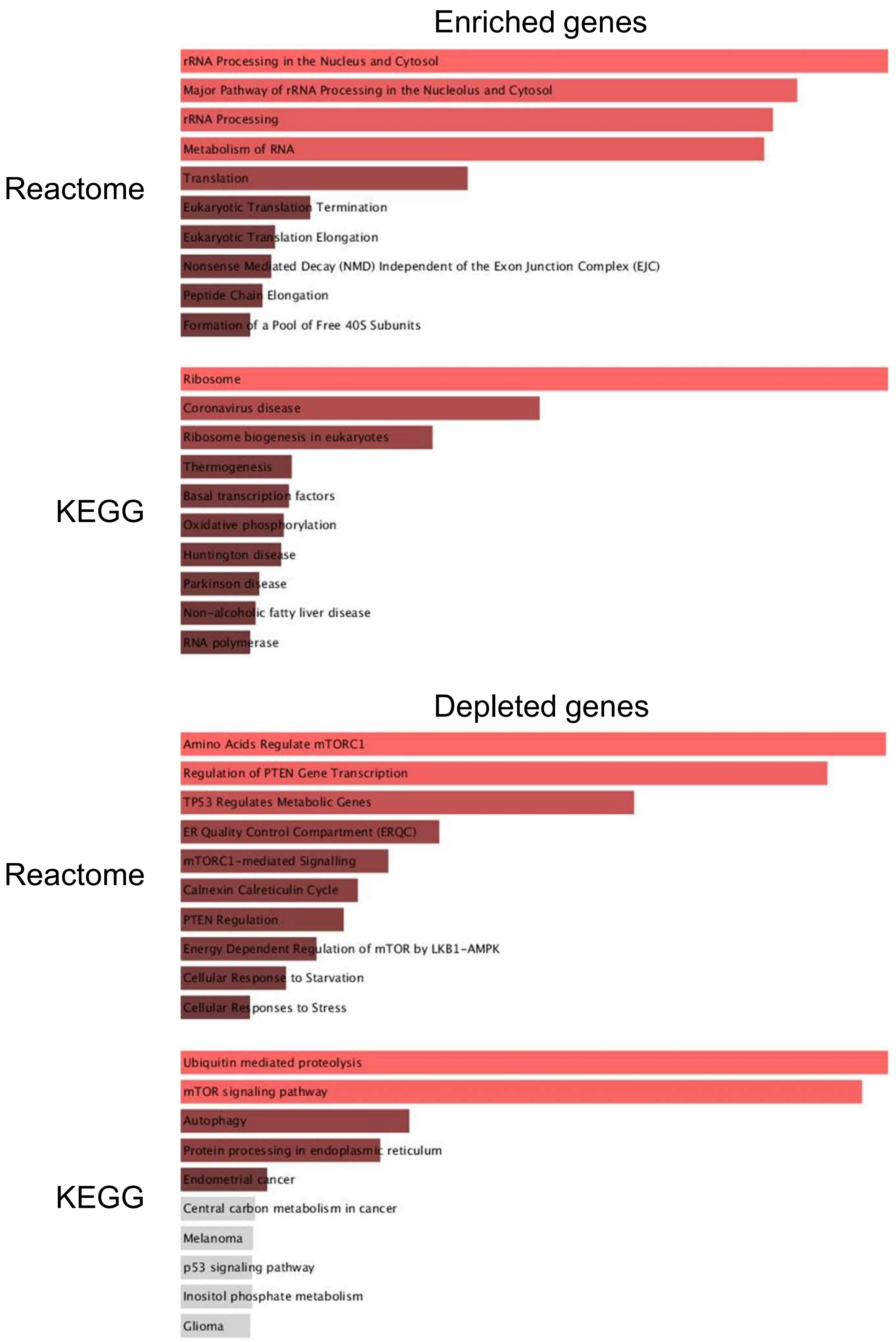

Supplementary Figure S2

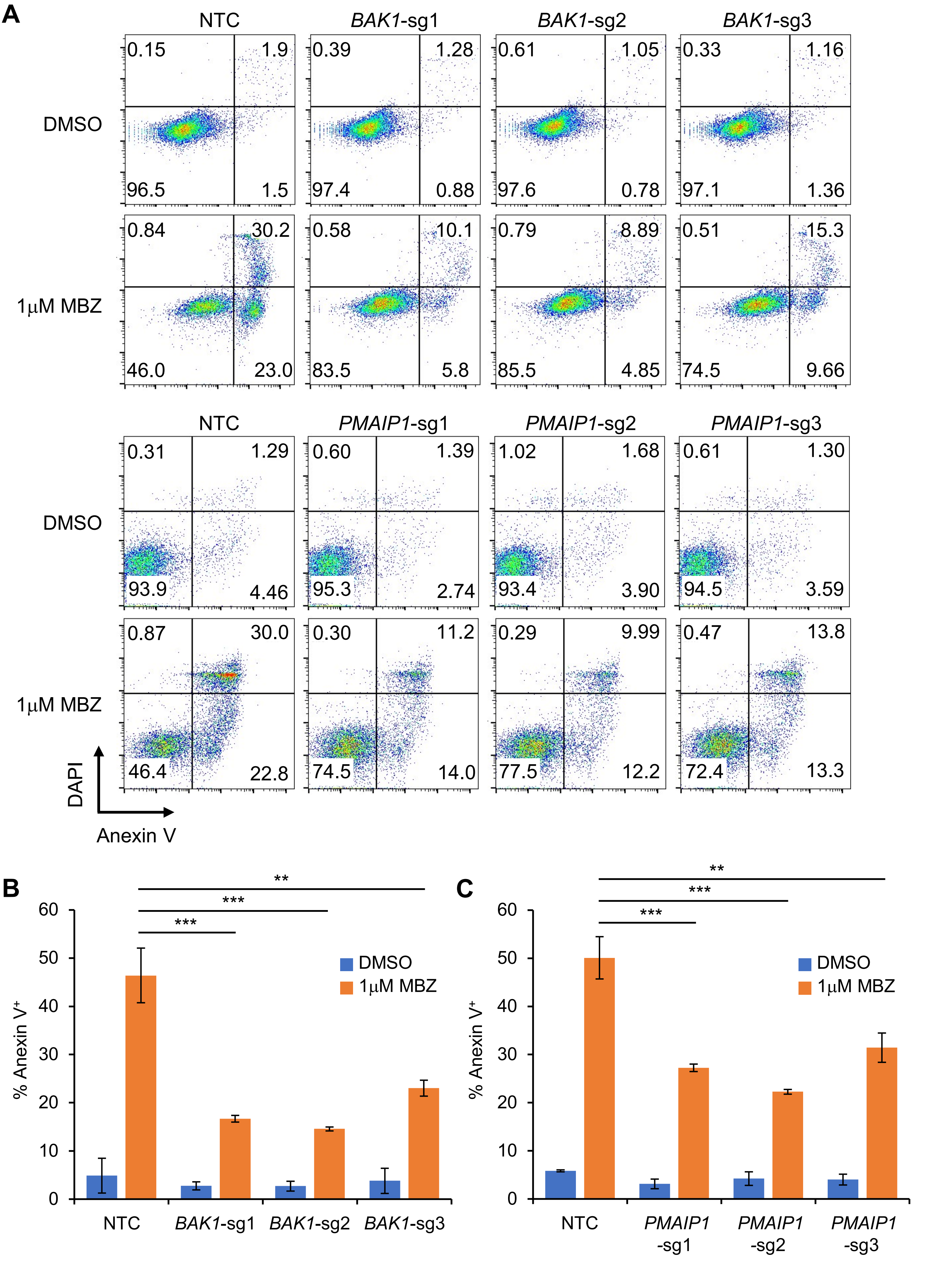

Supplementary Figure S3

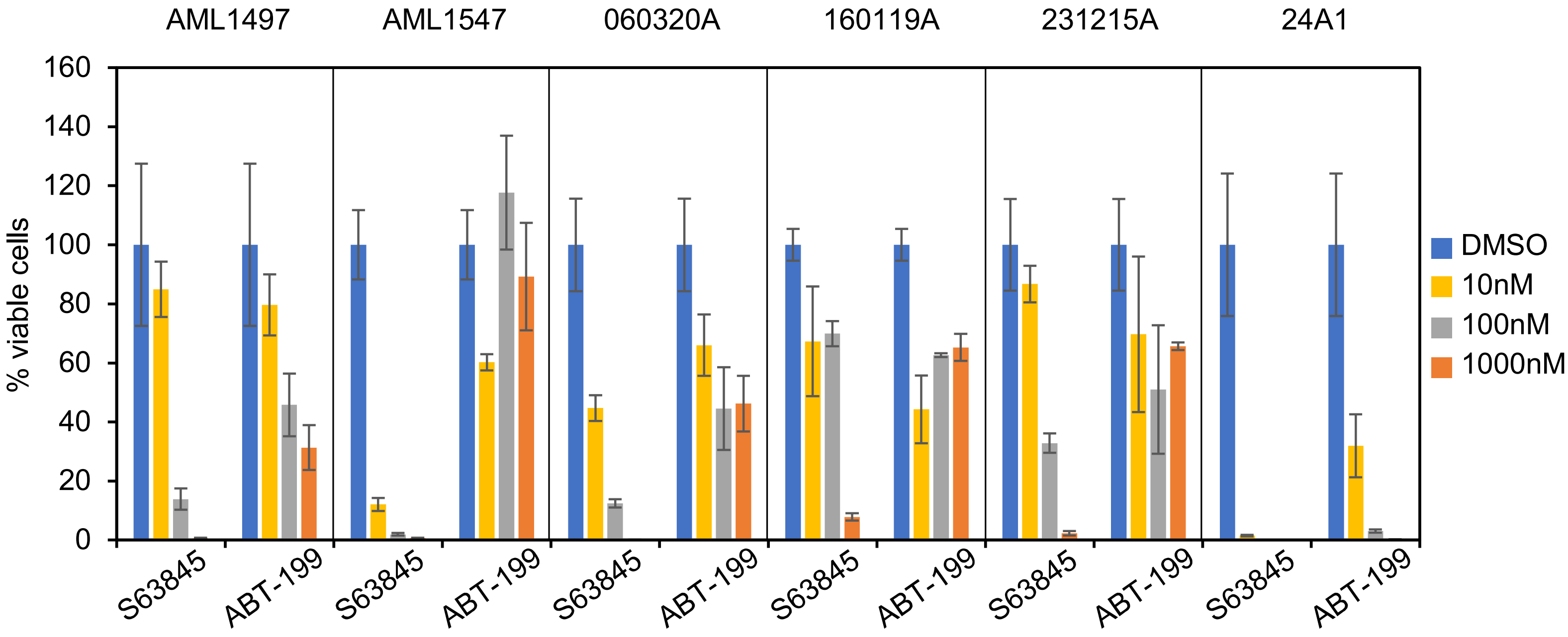

Supplementary Figure S4

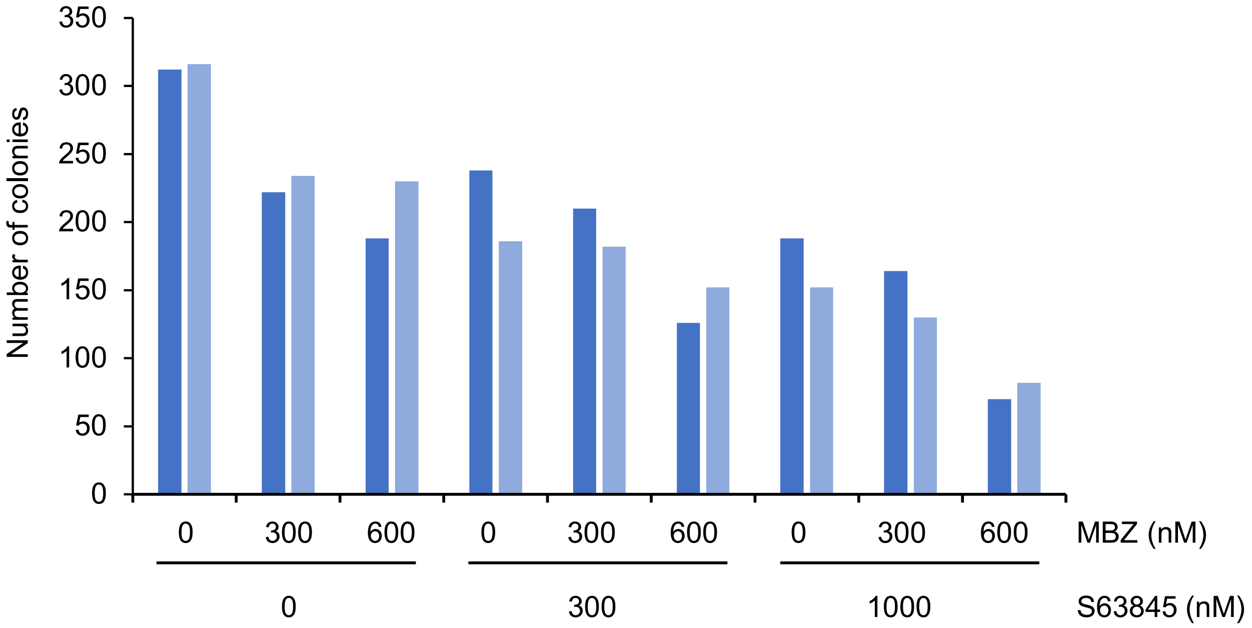

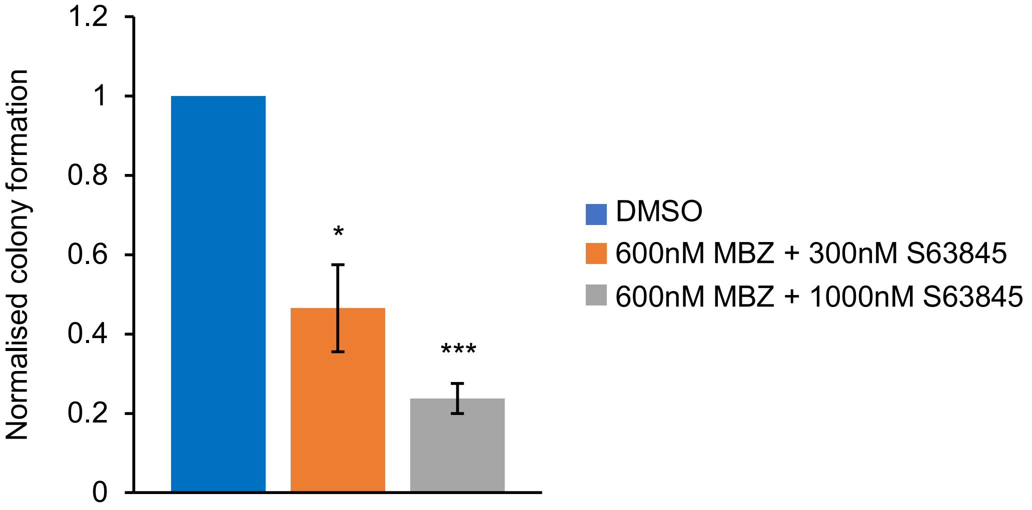

Supplementary Figure S5

Supplementary Figure Legends

Supplementary Fig. S1. Expression of BCL-2 and MCL1 following mebendazole exposure. **A** qRT-PCR analysis of BCL-2 gene expression in THP-1 cells 24 hours after treatment with 10 μM mebendazole. Gene expression data are normalised to DMSO-treated cells. Bars and error bars are means and SD of n=3 independent experiments. ***$P < 0.001$, two-tailed one sample *t* test. **B-C** Example of Western blots (left panel) and quantification (right panel) of **B** BCL-2 and **C** MCL1 protein expression in THP-1 cells exposed to 10 μM mebendazole for 24 hours, normalized to Tubulin loading control and DMSO treated controls. Bars and error bars are means and SD of n=3 independent experiments. *$P < 0.05$, two-tailed one sample *t* test.

Supplementary Fig. S2. Pathway analysis of positively and negatively enriched genes (FDR < 0.05) from the mebendazole CRISPR-Cas9 dropout screen. Enrichment was performed using the web tools found at https://maayanlab.cloud/Enrichr/

Supplementary Fig. S3. *BAK1* and *PMAIP1* knockout impair mebendazole induced apoptosis in THP-1 cells. **A** Examples of flow cytometry plots of THP-1 cells transduced with non-targeting control, *BAK1*-specific (upper panels) or *PMAIP1*-specific (lower panels) sgRNA following 48 h exposure to DMSO or 1 μM mebendazole (MBZ). Numbers in plots represent the percentage of cells within each quadrant. **B-C** Quantification of cell death induced by MBZ. Bars and error bars are means and SD of n=3 independent experiments. **$P < 0.01$; ***$P < 0.001$, two-tailed unpaired

Student's *t* test between MBZ-treated NTC and *BAK1* or *PMAIP1* sgRNA transduced cells.

Supplementary Fig. S4. PDX AML samples are more sensitive to MCL1 than BCL-2 inhibition. Plot shows viability of PDX AML cells, grown on OP9 monolayers, following 5 days exposure to indicated concentrations of S63845 or ABT-199.

Supplementary Fig. S5. The effect of mebendazole and S63845 on normal hematopoietic colony formation. **A** The graph shows the number of colonies formed by normal human $CD34^+CD133^+$ cord blood cells in methylcellulose in the presence of the indicated drug concentrations (n=1). Values from duplicate cultures are shown. **B** The graph shows the number of colonies formed by normal human $CD34^+CD133^+$ from 3 independent pooled cord blood samples in the presence of the indicated drug combinations, normalised to DMSO controls. Bars and error bars are means and SD of n=3 independent experiments. *$P < 0.05$; ***$P < 0.001$, two-tailed one sample *t* test.

Supplementary Materials and Methods

Cell culture

All AML cell lines were cultured in RPMI (Sigma-Aldrich), supplemented with 10% heat-inactivated FBS, penicillin-streptomycin (100µg/ml) and L-glutamine (2mM) (all Sigma-Aldrich) with the exception of SHI-1 cells which were cultured in IMDM supplemented with 20% heat-inactivated FBS, penicillin-streptomycin (100µg/ml) and L-glutamine (2mM) (all Sigma-Aldrich), in a humidified chamber with 5% CO2 at 37C. Each cell line was sub-cultured every 3-4 days and plated according to supplier guidelines. HEK293FT (ThermoFisher Scientific, Hemel Hempstead, UK) cells were maintained in DMEM with 10% heat-inactivated FBS, L-glutamine, penicillin/streptomycin and 500 µg/ml G418. The mouse OP9 cell line was grown in αMEM, supplemented with 20% heat-inactivated FBS, penicillin-streptomycin (100µg/ml) and L-glutamine (2mM).

OP9 co-cultures

OP9 cells were seeded into 96-well plates at a density of 20,000 cells per well and 24 hours later PDX AML cells were thawed and seeded onto OP9 monolayers at a density of 10,000-25,000 cells per well, in IMDM supplemented with 20% heat-inactivated FBS, penicillin-streptomycin (100µg/ml) and L-glutamine (2mM) (all Sigma-Aldrich), 50 ng/ml SCF and FLT3L, 20 ng/ml IL-3, IL-6, G-CSF and GM-CSF (all growth factors from PeproTech, London, UK). Indicated concentrations of mebendazole, S63845 or DMSO were then added to the co-cultures and incubated for 5 days. PDX AML cells were harvested for flow cytometric analysis gently without disrupting the OP9 monolayer.

Colony formation assays

Colony formation by normal human $CD34^{+}CD133^{+}$ cord blood-derived cells (ZenBio, NC, USA) was performed in HSC005 methylcellulose (Bio Techne, Abingdon, UK). Colony number was determined after 14 days culture.

In vivo transplantation

Group sizes were chosen based on previous estimates of disease latency in transplanted mice and experiments in the literature performing similar studies. No samples or animals were excluded from analysis.

Flow Cytometry

PDX AML cells were stained in 96 well plates with anti-human CD45 (clone H130, Biolegend UK, London, UK) and DAPI (Thermo Fisher Scientific) and analysed on the BD FACSymphony A5 using a BD High Throughput Sampler (BD Biosciences, Berkshire, UK). THP-1 cell apoptosis was determined by staining cells using the Annexin V Apoptosis Detection Kit APC (Thermo Fisher Scientific) in combination with DAPI. Mouse peripheral blood was stained with anti-mouse CD45 (clone 30-F11, Tonbo Biosciences, CA, USA) and anti-human CD45 and mouse erythrocytes lysed with eBioscience RBC Lysis Buffer (Thermo Fisher Scientific). EGFP expressing sgRNA transduced THP-1 cells were stained with TO-PRO-3 (Thermo Fisher Scientific). Stained cells were analysed on a LSRII analyser (BD Biosciences). Data was analysed using FlowJo v10 Software (BD Biosciences).

Synergy viability assays

Viability in synergy experiments for KASUMI-1, U937, NOMO-1, OCI-AML3, KCL22, SHI-1 and MV4;11 AML cell lines was assessed 72 h after drug exposure with the CellTiter 96 AQueous One Solution Cell Proliferation Assay (Promega, Southampton, UK), and for THP-1 cells by flow cytometry with the TO-PRO-3 (Thermo Fisher Scientific) viability dye.

Lentiviral transduction of human cell lines

293FT packaging cells (Thermo Fisher Scientific) were transiently co-transfected with the lentiviral expression vectors, the pCMV-PAX2 construct and the pVSV-G envelope construct (kind gifts of Prof D. Trono, Lausanne, Switzerland). Human leukemia cells were transduced with lentiviral supernatant by spinoculation at 700g, 25°C for 45 minutes in the presence of 5 μg/ml polybrene. CRISPR-Cas9 mediated knockout in THP-1 cells was performed with sgRNA targeting *BAK1* (sg1: BruLib1581-AAGACCCTTACCAGAAGCAG; sg2: BruLib1582-ACGGCAGCTCGCCATCATCG; sg3: BruLib1583-GCTCACCTGCTAGGTTGCAG) or *PMAIP1* (sg1: BruLib14241-CGCTCAACCGAGCCCCGCGC; sg2: BruLib14242-TCGAGTGTGCTACTCAACTC; sg3: BruLib14243-TTCTTGCGCGCCTTCTTCCC). The non-targeting control (NTC) sgRNA was (BruLib77289-TCAGTATCGGCTGCTGGTAA). sgRNA were cloned into the pL-CRISPR.SFFV.GFP (Addgene plasmid #57827) [1].

CRISPR drop-out screen

The mebendazole CRISPR drop-out screen was performed using the Human sgRNA library Brunello (addgene # 73179) [2] in lentiCRISPRv2 (addgene #52961) [3]. 500 x 10^6 THP-1 cells were transduced with the pooled sgRNA library, 2 μg/ml puromycin added after 48 h. Multiplicity of infection was calculated after 4 days puromycin selection, at which point the culture contained 29% viable cells by flow cytometry. Cells

were selected for 10 days in puromycin after which either 1 μM mebendazole or DMSO was added to the cultures and 10 days later viable cells enriched by density centrifugation using Lymphopure (BioLegend) and genomic DNA isolated using the QIAamp DNA Blood Maxi Kit (Qiagen, Manchester, UK). 50 x 10^6 and 16 x 10^6 cells were recovered for the DMSO and mebendazole conditions, respectively. The library was produced using forward and reverse barcode primers and the NEBNext High Fidelity PCR Master Mix (New England Biolabs, Hitchin, UK) according to previously published protocols [4] and pooled PCR products purified using the QIAquick PCR Purification Kit (Qiagen). Quality controls and quantification were performed on each library using the Qubit High Sensitivity DNA assay (Life Technologies) and the D1000 High Sensitivity ScreenTape on the TapeStation 4200 (Agilent, Santa Clara, CA, USA). Samples were equimolar pooled before 4 nM was denatured and diluted to the final running concentration of 1.8 pM using HT1 Buffer. The denatured and diluted pool was loaded onto the Illumina NextSeq 500 using a 75 cycle High Output reagent cartridge and flow cell, according to the manufacturer's instructions. Libraries were sequenced with an 84-cycle single read, plus an 8-cycle single index read. BCL output from the sequencer were converted to FASTQ, along with index demultiplexing using Illumina's BCL Convert v3.7.5. sgRNA enrichment was analysed using the open-source Galaxy platform (https://usegalaxy.org/). Briefly, 5' adapters were trimmed from the FASTQ files using the Cutadapt tool, sgRNA read counts were collected using the MAGeCK count tool and MAGeCKs test was used to perform sgRNA and gene ranking. Complexity of the library was maintained in both DMSO and mebendazole treated groups, Gini Index of 0.086 and 0.073, respectively. Percentage of mapped reads was 89.55 % and 90.89 %, and zero count sgRNA were 278 and 204, for DMSO and mebendazole treated groups, respectively, out of 77,441 total.

Western blot and immunoprecipitation (IP) analysis

The following primary antibodies were used: anti-total BAK (D4E4) and anti-MCL1 (D2W9E) (all Cell Signaling Technology), anti-active BAK (Ab-1, Merck Life Science), anti-MYB (05-175, Merck Life Science) and anti-GAPDH (sc-32233, Santa Cruz Biotechnology). IP analysis in THP-1 cells was performed using the Pierce™ Classic Magnetic IP/Co-IP Kit (ThermoFisher Scientific) according to manufacturer's instructions, but using a CHAPS buffer (1% CHAPS, 40 mM HEPES pH 7.4, 120 mM NaCl, 1 mM EDTA supplemented with protease and phosphatase inhibitors) for cell lysis and washes. 5 μl of anti-active BAK (Ab-1) was used in each immunoprecipitation reaction. Protein samples were resolved on 12.5 % polyacrylamide gels (0.36 M bis-Tris, 8-10 % acrylamide/bis) in MOPS-SDS running buffer (50 mM Tris, 50 mM MOPS, 1 mM EDTA, 0.1 % SDS). Gels were transferred onto nitrocellulose (LI-COR Biosciences, Cambridge, UK) membranes. Proteins were detected using appropriate IRDye 800CW and IRDye 680RD labelled secondary antibodies (LI-COR Biosciences). Quantification was performed on fluorescent images using the Odyssey® CLx and Image Studio software (LI-COR Biosciences).

References

1. Heckl D, Kowalczyk MS, Yudovich D, Belizaire R, Puram RV, McConkey ME, et al. Generation of mouse models of myeloid malignancy with combinatorial genetic lesions using CRISPR-Cas9 genome editing. Nat Biotechnol. 2014;32:941-946.

2. Doench JG, Fusi N, Sullender M, Hegde M, Vaimberg EW, Donovan KF, et al. Optimized sgRNA design to maximize activity and minimize off-target effects of CRISPR-Cas9. Nat Biotechnol. 2016;34:184-191.

3. Sanjana NE, Shalem O, Zhang F. Improved vectors and genome-wide libraries for CRISPR screening. Nat Methods. 2014;11:783-784.

4. Joung J, Konermann S, Gootenberg JS, Abudayyeh OO, Platt RJ, Brigham MD, et al. Genome-scale CRISPR-Cas9 knockout and transcriptional activation screening. Nat Protoc. 2017;12:828-863.

Supplementary Table S2. PDX AML characteristics.

PDX id	Sex	Age (yrs)	Patient Sample Cytogenetics
231215A	F	3.56	t(11;19)(q23;p13.1), *KMT2A-MLLT1*
24A1	M	12	t(11;19), *KMT2A-MLLT1*
060320A		0.66	t(10;11), *KMT2A-MLLT10*
160119A		0.44	Monosomy 7 detected by interphase FISH and G-band analysis.

AML1497 (*KMT2A-MLLT3*) and AML1547 (*KMT2A-MLLT3*) patient sample characteristics previously reported in Walf-Vorderwulbecke et al (2018). PDX samples were used at passage 1 (060320A), 2(24A1, AML1497, 160119A), 3 (231215A) or 4 (AML1547).
